# Sensory Processing, Functional Performance and Quality of Life in Unilateral Cerebral Palsy Children: A Cross-Sectional Study

**DOI:** 10.3390/ijerph17197116

**Published:** 2020-09-28

**Authors:** Patricia Jovellar-Isiegas, Inés Resa Collados, Diego Jaén-Carrillo, Luis Enrique Roche-Seruendo, César Cuesta García

**Affiliations:** 1 Faculty of Health Sciences, Universidad San Jorge, Campus Universitario, Autov A23 km 299, Villanueva de Gállego, 50830 Zaragoza, Spain; inesresa2@gmail.com (I.R.C.); djaen@usj.es (D.J.-C.); leroche@usj.es (L.E.R.-S.); 2 Department of Occupational Therapy, Faculty of Health Sciences, Centre for Advanced University Studies La Salle, Autonomous University of Madrid, 28023 Madrid, Spain; cesar.cuesta@lasallecampus.es; 3 Occupational Thinks Research Group, Institute of Neurosciences and Movement Sciences, Centre for Advanced University Studies La Salle, Autonomous University of Madrid, 28023 Madrid, Spain

**Keywords:** unilateral cerebral palsy, sensory processing, functional performance, activities of daily living, participation, quality of life

## Abstract

Background: The study of children with unilateral cerebral palsy (UCP) has traditionally focused on motor aspects. The extent to which sensory processing disorders can affect their functional performance and quality of life (QoL) is uncertain. This study aimed to explore the differences in sensory processing between UCP and typical development (TD) children and to analyze the relationship of sensory processing with functional performance and QoL. Methods: Fifty-three children aged from 6 to 15 years (TD = 24; UCP = 29) were recruited. The Child Sensory Profile 2, Pediatric Evaluation of Disability Inventory—Computer Adaptive Test and Kidscreen were used to evaluate sensory processing, functional performance and QoL. Results: UCP children showed sensory processing difficulties (avoidance: *p* = 0.02; registration: *p* = 0.00; body position: *p* = 0.00; oral: *p* = 0.02; social-emotional: *p* = 0.01), and scored lower in functional performance (daily activities: *p* = 0.00; mobility: *p* = 0.00; social/cognitive: *p* = 0.04) and in physical well-being (*p* = 0.00). The highest correlations in UCP group were found between proprioceptive processing and daily activities and mobility (r = −0.39); auditory, visual and tactile information and school environment (r = −0.63; r = −0.51; r = −0.46); behavioral and social-emotional responses and psychological well-being (r = −0.64; r = −0.49). Conclusions: UCP children have greater difficulty in sensory processing than TD children. Difficulties in proprioceptive processing contribute to poorer functional performance. Auditory, visual and tactile processing is associated with participation in the school environment and behavioral and social-emotional responses related to sensory processing are associated with the psychological well-being.

## 1. Introduction

Unilateral cerebral palsy (UCP) affects approximately 30% of children with cerebral palsy (CP) [1]. UCP children have mainly unilateral impairments as a result of a brain injury at either the fetal or infancy stage, usually located in the periventricular white matter, cortex and subcortex [2,3]. Usually, the upper limb (UL) is more affected than the lower limb, being considered as the main limitation for children’s participation in activities of daily living (ADL), which can also affect their quality of life (QoL) [4,5].

According to the International Classification of Functioning, Disability and Health for Children and Youth (ICF-CY) [6], valid and reliable activity-level outcome measures are often used for children with UCP; not only those measuring UL capacity, but also motor performance [7,8,9]. However, there are few instruments appropriate to measure children’s participation [10]. Children’s capacities do not always correspond to the way they perform in daily life [11]. Apparently, the functional performance in ADL of UCP children is below the age-matched normative data [12]. However, there are a few studies aimed to analyze functional performance and participation of UCP children and the degree of contribution of further factors, regardless of motor aspects.

The quality of the sensory input provides adequate feedback and guidance needed to correct the error in a motor action, being essential for successful motor task performance [13,14]. UCP children experience a variety of sensory inputs in a limited way due to impaired muscle tone, asymmetrical posture and improper postural adjustments and movement patterns [15]. This, added to the possible effect on the development of cortical and thalamic regions responsible for sensory processing of white substance destruction [16], suggests that sensory impairments can contribute to the functional problems of these children [17].

Sensory processing is a neurobiological process involving the registration and modulation of sensory information by neural systems. Moreover, the sensory input coming from the environment or the body itself has to be organized internally and integrated appropriately, thus the person can deliver a successful and adapted response to the demands of the environment. This allows the person to perform functional abilities of daily living and to engage in meaningful occupations [18]. The link established between cognition, behavior, participation and sensory processing has been suggested [19].

Different terminology can be found in relation to sensory processing. Dunn developed a theoretical model, Dunn’s sensory processing framework (DSPF) [20], which proposes an interaction between two continuums, the neurological threshold and behavioral self-regulation, to explain children’s performance. The neurological threshold refers to how rapidly people respond to sensory information, from a high to a low threshold. Behavioral self-regulation refers to how they manage sensory stimuli, ranging from passive to active behavior. These two continuums interact to create four sensory processing patterns [21]. According to the ICF-CY, DSPF focuses mainly on the interactions that are established between the person and the environment [6]. The knowledge about how UCP children process sensory inputs can provide information on how some aspects of activity and environmental demands can facilitate or hinder UCP children’s participation [20].

The sensory processing patterns proposed by Dunn are present in children with developmental conditions, such as autism spectrum disorder (ASD) and attention deficit and hyperactivity (ADH), being more likely to experience some challenges in occupational performance [22]. Although, these patterns have also been found in typical development (TD) children as a reflections of children’s abilities to respond to environmental demands [23]. Regarding CP children, it is known that there are disturbances in the way they process sensations [24], but, probably because these sensory disorders are masked by motor impairments [25], there is a gap in the literature and few studies have addressed this issue. Both motor and sensory impairments can have an impact in their QoL, which tends to be reported as lower compared to TD children [26,27,28]. However, the relationship between impairments and QoL is still uncertain and it seems that this relationship depends on the areas of life considered [29,30,31].

Greater and more comprehensive knowledge is needed about how UCP children handle sensory information and the relationship between this processing and the functional performance, participation and QoL. This knowledge can provide important insights into the adaptations of both activities and environmental demands to promote the participation of UCP children with families and practitioners.

Therefore, the aims of this study were (a) to describe the sensory processing, functional performance and QoL of UCP children compared to TD children and (b) to analyze the relationships between sensory processing, functional performance and QoL in both UCP and TD children.

## 2. Materials and Methods

### 2.1. Study Design

A cross-sectional study was performed. The study agreed with the STROBE statement [32]. The study was approved by the Ethics Committee for research with human beings, their data and samples from the La Salle Higher Center for University Studies (code CSEULS-PI-020/2019) and was carried out according with the Helsinki Declaration [33] and the current data protection law.

### 2.2. Participants and Procedures

Fifty-three children (29 in the case group and 24 in the control group) took part in the present study. Participants were selected using non-probabilistic convenience sampling and they were recruited in the Region of Aragon and in the Region of Madrid, Spain. The groups of cases were recruited from AIDIMO (Association for Research in Motor Disability) and Hemiweb (Childhood Hemiparesis Association). Both entities sent an explanatory letter via email to their databases. In the control group, the sample selection method was convenience type, including professional and personal contacts from the context of the researchers being associated in pairs to the cases by age and sex. Family socio-economic factors were not taken into account in the pairing. In order to recruit the control sample, the project was disseminated through an explanatory document via email. Those families who volunteered to participate and met the inclusion criteria signed an informed consent prior to participation. All the participants were recruited and assessed between October 2019 and February 2020, with the assessments being done on two different days so that the possible fatigue of the families when answering the questionnaires would not influence the results. On the first day, a therapist experienced with the use of the measurement tools explained how to complete the Child Sensory Profile 2 to the families. On the second day, the Pediatric Evaluation of Disability Inventory—Computer Adaptive Test and Kidscreen questionnaire (proxy and self-report versions) were completed.

The inclusion criteria for the UCP group were as follows: UCP diagnosis (congenital or acquired); aged between 6 and 15 years; Manual Ability Classification System (MACS) [34] level I-III; Gross Motor Classification System (GMFCS) [35] Level I-II; no botulinum toxin infiltration 4 months before the assessment; no moderate or severe cognitive impairment compatible with attending a special education school. The inclusion criteria for children in the TD group were as follows: no history of neurological disorder; aged between 6 and 15; no sensory processing disorder diagnosed by an occupational therapist; no occupational therapy or physiotherapy treatment. Additionally, all the children who presented fractures and/or trauma in the UL in the last 12 months were excluded. Criteria for abandonment were voluntary withdrawal by families or refusal to complete any of the questionnaires.

### 2.3. Variables and Data Measurements

#### 2.3.1. Child Sensory Profile 2 (CSP-2), Spanish Version

CSP-2 is an 86-item parent-report measure of a child’s sensory processing characteristics. The CSP-2 measures four sensory processing patterns based on quadrants of DSPF [20]: seeking, avoiding, sensitivity and registration. Additionally, the CSP-2 measures children’s responses to everyday events in six sensory domains (auditory, visual, touch, movement, body position and oral), and three behaviors related to sensory processing (behavioral, socio-emotional and attentional). For each item, parents were asked to describe their child’s response to a sensory experience on a 5-point Likert scale ranging from “0 = not applicable” and “1 = almost never or never” to “5 = almost always or always”. Higher scores represent higher frequency of behaviors, and then they are associated with higher sensory dysfunction. The Spanish version ensures the validity and reliability of the test in Spanish culture [36].

#### 2.3.2. Pediatric Evaluation of Disability Inventory—Computer Adaptive Test (PEDI-CAT)

The PEDI-CAT is a clinical assessment for children and youth from birth to 20 years of age and can be used across all diagnoses, conditions and settings. It was developed in 2011 as a revision and updated version of the PEDI and it was designed to be a parent-report measure to examine the functional performance of children across four domains: (1) daily activities; (2) mobility; (3) social/cognitive, and (4) responsibility [37].

The PEDI-CAT conceptual model was developed according to the ICF-CY framework, and activities and participation are strongly represented by the PEDI-CAT [38]. The activity ICF-CY domain is addressed in the daily activities, mobility and social/cognitive PEDI-CAT domains, and the participation ICF-CY domain is addressed in responsibility PEDI-CAT domain. The environment is also considered, including use of adaptations or modifications in the routines and defining the relevant context of performance within each item in the PEDI-CAT [37].

The PEDI-CAT has shown good reliability, validity and responsiveness in rehabilitation environments [39,40]. Furthermore, in children with CP, the PEDI-CAT has demonstrated to be a valid instrument for functional ability measurement [41,42].

#### 2.3.3. Kidscreen Questionnaire, Spanish Version

The Kidscreen questionnaire measures health-related quality of life (HRQoL) in healthy and chronically ill children from 8 to 18 years old [43]. This instrument was developed in a European project in which 13 countries were involved [44] and self- and proxy-report versions are available. In the present study, Kidscreen-27 proxy version (KS-27) was used to measure parental perception of children’s health and wellbeing across 5 domains: physical well-being, psychological well-being, autonomy and parent relations, social support and peers and school environment. KS-27 is a short version of Kidscreen-52 which has been used to assess HRQoL in children who have suffered from stroke [45]. This instrument can be implemented equally in children with CP and in the general population [46].

Additionally, the Kidscreen-10 self-report version (KS-10) was included in this study to assess children’s perceptions of their global HRQoL, including physical, psychological and social aspects of HRQoL. Conceptually and psychometrically, KS-10 has demonstrated to be a good option for CP children [47].

### 2.4. Sample Size

The sample size was calculated using G*Power (v3.1.9.2, Heinrich-Heine-University, Dusseldorf, Germany). Expecting a correlation from moderate to large (0.3 to 0.7) between questionnaires, with an alpha value of 0.05 and a beta value of 0.1 (power 90%), the a priori calculation for a two-tails bivariate correlation analysis indicated that a sample of 36 participants was needed. This sample size was enough to detect an *r* effect size of 0.45 between cases and controls in the domains of the CSP-2 for a one-tail difference between a two-group analysis (alpha value of 0.05, beta value of 0.2). Previous research comparing cases and controls reported an *r* effect size of 0.6 [24]; however, we expected to observe smaller differences between groups due to the expected level of disability in our sample of cases, which would be smaller.

### 2.5. Statistical Methods

Data analysis was conducted using SPSS (vs. 25, SPSS Inc., Chicago, IL, USA). Data were examined for normality using the Shapiro–Wilk test. Descriptive statistics are represented as means and standard deviation (SD). In order to compare the sensory processing, functional performance and QoL between UCP and TD children, a comparison of means was conducted (using a *t* test for parametric or Mann–Whitney test for non-parametric quantitative variables, and a Chi-squared test for qualitative variables). Depending on parametric or non-parametric data distribution, Cohen’s d effect size and r, respectively, were used to interpret the magnitude of the differences (i.e., an effect size of less than 0.2 reflects a negligible mean difference; between 0.2 and 0.5, a small difference; between 0.5 and 0.8, a moderate mean difference; 0.8 or greater, a large difference) [48]. A Pearson correlation analysis was also conducted. To properly interpret the magnitude of correlations, the following criteria were adopted: <0.1 (trivial), 0.1–0.3 (small), 0.3–0.5 (moderate), 0.5–0.7 (large), 0.7–0.9 (very large) and 0.9–1.0 (almost perfect) [49]. The level of significance used was *p* < 0.05.

## 3. Results

### 3.1. Characteristics of the Sample

As can be seen in Table 1, a total of 53 children with a mean age of 9.51 (SD ± 4.47) participated in the study. All the participants completed the CSP-2, but only 49 (TD = 23; UCP = 26) completed the PEDI-CAT because they did not attend the second day of the evaluation. The Kidscreen questionnaire was completed by 36 families and children (TD = 16; UCP = 20), designed for 8-year-old children and older. No statistically significant differences were found between the TD and UCP children, neither in age nor in gender. There was a significantly higher proportion of right-handed children in the TD group than in the UCP group due to the distribution of the damage (55.2% right UCP).

### 3.2. Differences in Sensory Processing, Functional Performance and HRQoL between Groups

**Figure 1:**
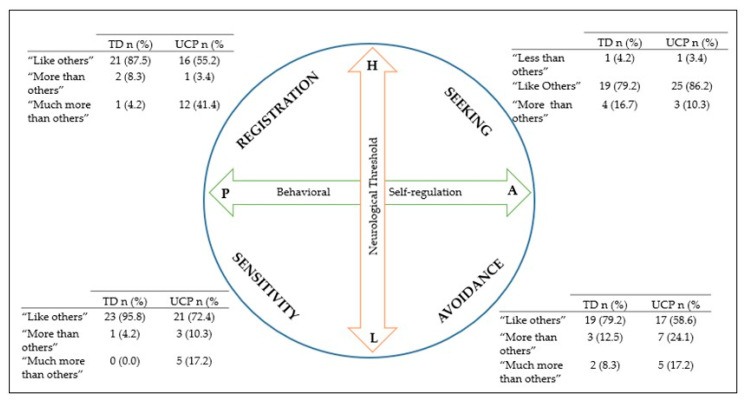
Sensory Profile Patterns distribution in TD and in UCP groups according to the normative data of the Child Sensory Profile 2. “Less than others”: −1 standard deviation below norm; “Like others”: within norm; “More than others”: +1 standard deviation above norm; “Much more than others”: +2 standard deviation above norm.

The statistical differences between groups found in CSP-2, PEDI_CAT, KS-27 and KS-10 can be observed in Table 2. Related to the Sensory Profile Patterns, the UCP group scores were higher than the TD group’s in the avoidance and registration profile, these differences being statistically significant (*p* = 0.02, *p* = 0.00, with a small (r = 0.32) and moderate effect size (r = 0.52), respectively) (Figure 1). Regarding the Sensory Systems, statistically significant differences were found in body position (*p* = 0.00) with a moderate effect size (r = 0.61) and oral (*p* = 0.02) with a small effect size (r = 0.32); and no statistically significant differences were found between groups in auditory, visual, touch and movement. Finally, in the social-emotional domains, the UCP group showed significantly higher scores as compared to the TD group (*p* = 0.01) with a small effect size (r = 0.33).

The PEDI-CAT showed statistically significant differences between groups in all domains, except in responsibility; especially in daily activities (*p* = 0.00), there was a large effect size (d = 2.21) and in mobility (*p* = 0.00) there was a moderate effect size (r = 0.70).

The KS-27 only showed a statistically significant difference in the physical well-being domain (*p* = 0.00) with a large effect size (d = 1.06). Additionally, a moderate effect size (d = 0.534) was found for the dimension of school environment, although the difference found was not statistically significant. Regarding the KS-10, no statistically significant differences between groups were found.

### 3.3. Correlations between Sensory Processing and Funcional Performance

Correlations showed a significantly negative moderate to large relationship between the avoidance and registration sensory profile patterns and daily activities and mobility domains (r = −0.41–−0.58). In the TD group, these relationships were maintained for daily activities but not for mobility. However, in the UCP group, they disappeared. In sensory systems, the highest significant correlations were found between body position and daily activities (r = −0.70), mobility (r = −0.67) and social/cognitive (r = 0.41) for the entire group. In the UCP group, these relationships were maintained for daily activities and mobility, although they were moderate and less significant. Regarding behaviors related to sensory processing, despite finding a significant negative relationship between social-emotional and daily activities (r = −0.46) and mobility (r = −0.44) for the entire group, no significant relationship was found in the UCP group (Supplementary Table S1).

### 3.4. Correlations between Sensory Processing and HRQoL

The correlations between the CSP-2 and the Kidscreen-27 and -10 domains can be observed in Supplementary Table S2. In the UCP group, the highest significant correlations found in the sensory systems can be seen between the auditory (r = −0.63), visual (r = −0.51), touch (r = −0.46) and school environment domains. Except for the tactile system, this was maintained when the entire group was analyzed, the relationship being the most significant. A significant moderate to large correlation was found between social-emotional and parents and autonomy, both in the entire and in the TD group, although less significant in the latter. In the UCP group, a large, negatively significant correlation was found between the behavioral and psychological well-being domains (r = −0.64), and moderate between the social-emotional and psychological well-being domains (r = −0.49). Between CSP-2 and Kidscreen-10, no significant relationship was found in any of the groups analyzed.

## 4. Discussion

### 4.1. Sensory Processing, Functional Performance and QoL

To the authors’ knowledge, this is the first study aimed to analyze the sensory processing of UCP children compared to TD children and to examine its relationship with functional performance and QoL. Our results showed that UCP children present, more frequently, the avoidance and the registration sensory profile pattern compared to TD children. Significant differences were found in the body position, oral sensory systems and in the socio-emotional responses, showing that UCP children have greater difficulties in sensory processing than TD children, as well as presenting greater difficulty in functional performance related to daily activities, mobility and social/cognitive function. However, our results exhibited that the UCP children in this sample can take control over major life tasks that enable independent living. The HRQoL perceived by the UCP children’s families was similar to that perceived by TD children’s families, except in the physical well-being domain. The HRQoL perceived by the children was also similar in both groups. Although there was a higher proportion of girls in the UCP group in the sample of this study, no statistically significant differences were found between the two groups, so this was not considered to influence the results. There was a significantly higher proportion of right-handed children in the TD group than in the UCP group due to the distribution of damage, so dominance was not considered as a covariate in this study.

Little et al. [23] shows that there are different sensory subtypes present in both TD children and those with developmental conditions. Our results indicated that this also occurs in UCP children. IN total, 41.2% of UCP children had the avoider sensory profile pattern “more/much more” than other children. This means, according to Blanche et al. [25] and Pavão et al. [24], that their neurological threshold is low, thus they are too sensitive to certain sensory stimuli. In order to self-regulate this behavior, they engaged in active behavior to try to reduce sensory inputs. About 44.8% of UCP children had the sensory profile register “more/much more” than other children, which means that their neurological threshold is high, so they tend to ignore some stimuli and they self-regulate their behavior passively (i.e., remaining spectators). Due to the high neurological threshold of UCP children, they may react more slowly to certain stimuli. Therefore, they need these stimuli to be more intense in order to detect what is happening around them and to be able to engage in the activities they perform. This under-responsiveness coincides with that described by Blanche et al. [25], Soomro et al. [50] and Pavão et al. [24,51] in CP children.

Regarding sensory systems, our results revealed that UCP children process auditory (*p* = 0.14), visual (*p* = 0.80) and vestibular (*p* = 0.29) information in a way that is comparable to TD children. However, other studies, such as Prakash et al. [52] and Soomro et al. [50], reported difficulties in auditory and visual processing. The differences in the results can be explained in that the aforementioned studies included children with GMFCS levels I-V, while in our study there were only children in levels I and II. According to Park [53], the higher the gross motor capabilities, the better the sensory processing abilities. This also explains why Prakash et al. [52] and Pavão et al. [24] reported encountering difficulties in processing vestibular information. It is arguable that UCP children are more able to move freely and therefore have more experience and opportunities to be able to process the vestibular input properly, as Soomro et al. [50] found for UCP children.

Our results, according to Pavão et al. [24], indicated that UCP children can modulate tactile input (*p* = 0.39). It seems, however, that their difficulties are more related to the registration and discrimination phases. In fact, 40% of UCP children presented deficits in registration and perception of tactile information in the affected UL and 37% presented deficits only in perception [54].

UCP children have greater difficulty in modulating proprioceptive input (body position *p* = 0.00). This has also been reported by Prakash et al. [52] and Pavão et al. [24], and related to poor postural control in CP children [51]. The differences found in the oral system (*p* = 0.02) might be related to the prevalence of the avoidance profile in the UCP group, as these children often show avoidance responses related to certain foods and textures. Finally, our results indicated significant differences in the social–emotional responses related to sensory processing (*p* = 0.01) shown by the UCP group, which is common to all types of CP [24,52].

Similar to our findings, Van Zelst et al. [12] found that the functional performance of UCP children was below age-matched normative data, suggesting that process skill deficits were more important than motor skills by themselves. Østensjø et al. [55] found that CP children differed to a great extent from the normative sample of the PEDI in the daily activities, mobility and social/cognitive domains, similar to the present study. However, regarding participation (responsability PEDI-CAT domain *p* = 0.29), our results indicated that, even though they have functional performance problems, children with UCP participate equally to their TD peers. McManus et al. [56] and Michelsen et al. [57] agreed with this finding.

Of note, despite low scores on functional performance, our results indicated that children with UCP perceive their HRQoL in the same way as typically developing peers (*p* = 0.29). These results coincide with those found by Dickinson et al. [30] and Shelly et al. [58], challenging the assumption that when children have poor functioning, they necessarily have a lower QoL in all domains. These results can also be explained by the “disability paradox” [59], whereby people with disabilities can have a good QoL. Furthermore, our results show that families of children with UCP perceive the same QoL in relation to their children as families of TD children, similarly to what Shelly et al. [58] proposes, except in the physical well-being domain (*p* = 0.00). This makes sense considering the important motor consequences of the brain damage.

Contrary to our study, Russo et al. [27] found significant differences in HRQoL measured by the Pediatric Quality of Life Inventory (PedsQL), both in the proxy and in the self-report version. These differences between studies may be explained by the nature of the questionnaires being used. The Kidscreen questionnaire is a “general” instrument and, although it can be used in children with CP [46], it may not identify all the priorities that children with UCP have [60]. Since HRQoL is a multidimensional concept which is influenced by many personal, psychosocial and contextual factors, the only way to determine HRQoL is to assess it directly [61]; thus, in this sense, further research is needed in the UCP population.

### 4.2. Correlations between Sensory Processing and Functional Performance

When the entire group is analyzed, the patterns of sensory profiles are negatively associated with functional performance, especially the registry (ADL: r = −0.58 *p* < 0.001; mobility: r = −0.48 *p* < 0.001; social/cognitive: r = −0.34 *p* < 0.05), followed by the avoidance (ADL: r = −0.44 *p* < 0.01; mobility: r = −0.41 *p* < 0.01), and finally the sensitive profile (ADL: −0.34 *p* < 0.05; mobility: r = −0.32 *p* < 0.05; social/cognitive: r = −0.32 *p* < 0.05). The more children present these profiles, the worse they perform. Proprioceptive processing has a large negative relationship with this performance (ADL: r = −0.70 *p* < 0.001; mobility: r = −0.67 *p* < 0.001; social/cognitive: r = −0.41 *p* < 0.01) as well as the social-emotional responses (ADL: r = −0.46 *p* < 0.001; mobility: r = −0.44 *p* < 0.01), meaning that the more difficulties they have in processing sensory information, the worse their performance is. This confirms, as previously reported, that adequate sensory processing allows the person to perform functional abilities of daily living [18]. If these children, both TD and disabled (including UCP), have a sensory modulation disorder, they are more likely to experience some challenges in occupational performance [22,62,63].

In UCP children, the sense of body position has a negative association with functional performance of ADL (r = −0.39 *p* < 0.05) and Mobility (r = −0.39 *p* < 0.05). This highlights the importance of unconscious proprioceptive information in posture and anticipatory control, and how this can influence movement planning [64]. Other authors have reported interesting associations between different somatosensory aspects with both the motor capacity and the bimanual motor performance of the UL, but not with the functional performance of ADL [65,66,67,68,69,70,71]. Notwithstanding, these authors have identified deficits in registration and discrimination, but not in modulation. It would be advisable to include an assessment that involves all the sensory processing phases and evaluate to what extent this may affect the child’s functional performance.

### 4.3. Correlations between Sensory Processing and HRQoL

The processing of auditory and visual information negatively correlates with the HRQoL perceived by the families of the whole group regarding school environment (r = −0.53 *p* < 0.001 and r = −0.42 *p* < 0.01, respectively). This supports the idea that if children present sensory processing difficulties, this can affect the development of their executive functions [72,73]. Moreover, according to Hahn et al. [74], visual and auditory processing have a great impact on learning mechanisms. In the UCP group, additionally, tactile processing was also associated with the school environment (r = −0.46 *p* < 0.05). This could be explained by the association between sensory sensitivity (low threshold in the avoidant profile) and attention difficulties [75]. Finally, behavioral and socio-emotional responses negatively correlate with the psychological well-being of UCP children as perceived by their families (r = −0.64 *p* < 0.01 and r = −0.49 *p* < 0.05, respectively).

Our study is subject to some limitations. First, the sampling of participants was non-probabilistic. Therefore, generalizing the results to a large population may be difficult, and these are only applicable to UCP children without moderate or severe cognitive impairment. Second, the PEDI-CAT is translated but not validated for the Spanish population, thus, it may not be able to identify some limitations and/or restrictions in the participation of Spanish children. Third, although none of the TD children had a diagnosis of sensory processing disorder, they were not screened prior to the study, so some of them could have sensory processing issues to a certain extent. Ultimately, we have not included an assessment that would detect deficits in the somatosensory discrimination of the UL. Despite this, our study is the first to expose and clarify sensory processing disorders in UCP children. Therefore, future lines of research should deepen into analyzing somatosensory discrimination deficits in UL, including a complete somatosensory assessment battery given its important contribution to motor control, and examine both their relationship with functional performance and the influence that the affected side, age or gender may have on their prevalence.

## 5. Conclusions

Children with UCP have greater difficulty in sensory processing than TD children. The processing difficulties of proprioceptive input related to the handling of their body in space contribute to poorer functional performance. The processing of Auditory, Visual and Tactile information is associated with participation in the school environment and behavioral and social-emotional responses related to sensory processing are associated with the psychological well-being of UCP children. This study has clinical implications for both the families and practitioners, showing that knowing how UCP children process sensory information can provide important insights into the adaptations of both activities and environment demands to promote their participation.

## Figures and Tables

**Table 1:** Sociodemographic characteristics and hand dominance by group.

Characteristics	TD *n* (24)	UCP *n* (29)	*p* Value
Age, mean (SD) IQR	9.60 (2.30) [7.36–11.45]	9.44 (2.64) [6.83–10.11]	0.815 ^1^
Gender, *n* (%)			
Female	10 (41.7)	18 (62.1)	0.139 *^2^*
Male	14 (58.3)	11 (37.9)	
Hand dominance, n (%)			
Right	22 (91.7)	13 (44.8)	0.000 ^2^
Left	2 (8.3)	16 (55.2)	

TD: typical development; UCP: unilateral cerebral palsy; SD: standard deviation; IQR: interquartile range; ^1^ Student *t* test; ^2^ Chi-squared test.

**Table 2:** Differences in CSP-2, PEDI-CAT and Kidscreen-27 and -10 between groups.

Variables	TD	UCP		
	Mean (SD)	Mean (SD)	*p* Value	ES
CSP-2 Categories	*n* (24)	*n* (29)		
Seeking	1.88 (0.54)	1.83 (0.55)	0.76 ^1^	0.08 ^†^
Avoidance	1.72 (0.54)	2.09 (0.61)	0.02 ^2^	0.32 ^‡^
Sensitivity	1.50 (0.30)	1.84 (0.65)	0.09 ^2^	0.23 ^‡^
Registration	1.40 (0.54)	2.02 (0.71)	0.00 ^2^	0.52 ^‡^
Auditory	2.02 (0.70)	2.46 (0.97)	0.14 ^2^	0.20 ^‡^
Visual	2.13 (0.62)	2.08 (0.66)	0.80 ^1^	0.07 ^†^
Touch	1.28 (0.34)	1.39 (0.52)	0.39 ^2^	0.12 ^‡^
Movement	1.87 (0.49)	2.05 (0.66)	0.29 ^2^	0.15 ^‡^
Body Position	1.23 (0.34)	2.23 (0.93)	0.00 ^2^	0.61 ^‡^
Oral	1.46 (0.44)	1.93 (0.81)	0.02 ^2^	0.32 ^‡^
Behavioral	1.63 (0.55)	1.72 (0.57)	0.61 ^2^	0.07 ^‡^
Social-Emotional	1.69 (0.60)	2.15 (0.80)	0.01 ^2^	0.33 ^‡^
Attentional	1.71 (0.75)	1.98 (0.84)	0.16 ^2^	0.19 ^‡^
PEDI-CAT Domains	*n* (23)	*n* (26)		
Daily Activities	54.22 (10.14)	29.23 (12.25)	0.00 ^1^	2.21 ^†^
Mobility	56.87 (10.61)	30.65 (16.50)	0.00 ^2^	0.70 ^‡^
Social/Cognitive	51.65 (8.26)	45.15 (12.59)	0.04 ^1^	0.60 ^†^
Responsibility	50.30 (9.55)	47.08 (13.97)	0.29 ^2^	0.15 ^‡^
KIDSCREEN-27	*n* (16)	*n* (20)		
Physical Well-Being	53.38 (9.60)	43.40 (9.34)	0.00 ^1^	1.06 ^†^
Psychological Well-Being	50.01 (7.13)	51.33 (9.16)	0.64 ^1^	0.16 ^†^
Parents and Autonomy	50.06 (5.36)	55.26 (10.01)	0.10 ^2^	0.28 ^‡^
Social Support and Peers	55.36 (6.58)	55.86 (9.35)	0.86 ^1^	0.06 ^†^
School Environment	52.87 (7.32)	56.91 (7.73)	0.12 ^1^	0.53 ^†^
KIDSCREEN-10				
General HRQoL index	55.09 (9.11)	50.84 (6.96)	0.26 ^2^	0.19 ^‡^

CSP-2: Child Sensory Profile 2; PEDI-CAT: Pediatric Evaluation of Disability Inventory – Computer Adaptive Test; TD: typical development; UCP: unilateral cerebral palsy; SD: standard deviation; ES: effect size; HRQoL: health related quality of life; ^1^ Student *t* test; ^2^ Mann–Whitney U test; ^†^ Cohen´s d; ^‡^ r value.

## Supplementary Materials

The following are available online at https://www.mdpi.com/1660-4601/17/19/7116/s1, Table S1: Correlations between CSP-2 and PEDI-CAT domains, Table S2: Correlations between CSP-2 and KD-27 and KD-10 domains.

## References

[1] Stanley F.J., Blair E., Alberman E. (2000). Cerebral Palsies: Epidemiology and Causal Pathways.

[2] Cioni G., Sales B., Paolicelli P.B., Petacchi E., Scusa M.F., Canapicchi R. (1999). MRI and clinical characteristics of children with hemiplegic cerebral palsy. Neuropediatrics.

[3] Rosenbaum P., Paneth N., Leviton A., Goldstein M., Bax M., Damiano D., Dan B., Jacobsson B. (2007). A report: The definition and classification of cerebral palsy April 2006. Dev. Med. Child Neurol. Suppl..

[4] Sakzewski L., Ziviani J., Boyd R.N. (2014). Efficacy of upper limb therapies for unilateral cerebral palsy: A meta-analysis. Pediatrics.

[5] Fedrizzi E., Pagliano E., Andreucci E., Oleari G. (2003). Hand function in children with hemiplegic cerebral palsy: Prospective follow-up and functional outcome in adolescence. Dev. Med. Child Neurol..

[6] World Health Organization (2007). International Classification of Functioning, Disability, and Health: Children & Youth Version: ICF-CY.

[7] Klingels K., Jaspers E., Van de Winckel A., De Cock P., Molenaers G., Feys H. (2010). A systematic review of arm activity measures for children with hemiplegic cerebral palsy. Clin. Rehabil..

[8] Gilmore R., Sakzewski L., Boyd R. (2010). Upper limb activity measures for 5- to 16-year-old children with congenital hemiplegia: A systematic review. Dev. Med. Child Neurol..

[9] Lemmens R.J., Timmermans A.A., Janssen-Potten Y.J., Smeets R.J., Seelen H.A. (2012). Valid and reliable instruments for arm-hand assessment at ICF activity level in persons with hemiplegia: A systematic review. BMC Neurol..

[10] Morris C., Kurinczuk J.J., Fitzpatrick R. (2005). Child or family assessed measures of activity performance and participation for children with cerebral palsy: A structured review. Child Care Health Dev..

[11] Smits D.W., Gorter J.W., Ketelaar M., Van Schie P.E., Dallmeijer A.J., Lindeman E., Jongmans M.J.J.D.M., Neurology C. (2010). Relationship between gross motor capacity and daily-life mobility in children with cerebral palsy. Dev. Med. Child. Neurol..

[12] Van Zelst B., Miller M.D., Russo R.N., Murchland S., Crotty M.J.D.M., Neurology C. (2006). Activities of daily living in children with hemiplegic cerebral palsy: A cross-sectional evaluation using the assessment of motor and process skills. Dev. Med. Child. Neurol..

[13] Papadelis C., Ahtam B., Nazarova M., Nimec D., Snyder B., Grant P.E., Okada Y. (2014). Cortical somatosensory reorganization in children with spastic cerebral palsy: A multimodal neuroimaging study. Front. Hum. Neurosci..

[14] Dunn W., Daniels D.B.D. (2002). Initial development of the infant/toddler sensory profile. OTJR Occup. Particip. Health.

[15] Blanche E.I., Botticelli T.M., Hallway M.K. (1995). Combining Neuro-Developmental Treatment and Sensory Integration Principles: An Approach to Pediatric Therapy.

[16] Tsao H., Pannek K., Fiori S., Boyd R.N., Rose S. (2014). Reduced integrity of sensorimotor projections traversing the posterior limb of the internal capsule in children with congenital hemiparesis. Res. Dev. Disabil..

[17] Kuo H.-C., Gordon A.M., Henrionnet A., Hautfenne S., Friel K.M., Bleyenheuft Y. (2016). The effects of intensive bimanual training with and without tactile training on tactile function in children with unilateral spastic cerebral palsy: A pilot study. Res. Dev. Disab..

[18] Humphry R. (2002). Young Children’s occupations: Explicating the dynamics of developmental processes. Am. J. Occup. Ther..

[19] Dunn W., Little L., Dean E., Robertson S., Evans B. (2016). The State of the science on sensory factors and their impact on daily life for children: A scoping review. OTJR Occup. Particip. Health.

[20] Dunn W. (2014). Sensory Profile–2 User’s Manual.

[21] Dean E.E., Little L., Tomchek S., Dunn W. (2018). Sensory processing in the general population: Adaptability, resiliency, and challenging behavior. Am. J. Occup. Ther..

[22] Little L.M., Ausderau K., Sideris J., Baranek G.T. (2015). Activity participation and sensory features among children with autism spectrum disorders. J. Autism Dev. Disord..

[23] Little L.M., Dean E., Tomchek S.D., Dunn W. (2017). Classifying sensory profiles of children in the general population. Child Care Health Dev..

[24] Pavão S.L., Rocha N.A.C.F. (2017). Sensory processing disorders in children with cerebral palsy. Infant Behav. Dev..

[25] Blanche E., Nakasuji B. (2001). Understanding the Nature of Sensory Integration with Diverse Populations.

[26] Livingston M.H., Rosenbaum P.L., Russell D.J., Palisano R.J. (2007). Quality of life among adolescents with cerebral palsy: What does the literature tell us?. Dev. Med. Child Neurol..

[27] Russo R.N., Goodwin E.J., Miller M.D., Haan E.A., Connell T.M., Crotty M. (2008). Self-esteem, self-concept, and quality of life in children with hemiplegic cerebral palsy. J. Pediatr..

[28] Caspar-Teuscher M., Studer M., Regenyi M., Steinlin M., Grunt S., Swiss Neuropediatric Stroke Registry Group (2019). Health related quality of life and manual ability 5 years after neonatal ischemic stroke. Eur. J. Paediatr. Neurol..

[29] Natalucci G., Bucher H.U., Von Rhein M., Tolsa C.B., Latal B., Adams M. (2017). Population based report on health related quality of life in adolescents born very preterm. Early Hum. Dev..

[30] Dickinson H.O., Parkinson K.N., Ravens-Sieberer U., Schirripa G., Thyen U., Arnaud C., Beckung E., Fauconnier J., McManus V., Michelsen S.I. (2007). Self-reported quality of life of 8–12-year-old children with cerebral palsy: A cross-sectional European study. Lancet.

[31] Arnaud C., White-Koning M., Michelsen S.I., Parkes J., Parkinson K., Thyen U., Beckung E., Dickinson H.O., Fauconnier J., Marcelli M. (2008). Parent-reported quality of life of children with cerebral palsy in Europe. Pediatrics.

[32] Von Elm E., Altman D.G., Egger M., Pocock S.J., Gotzsche P.C., Vandenbroucke J.P., Initiative S. (2014). The Strengthening the Reporting of Observational Studies in Epidemiology (STROBE) Statement: Guidelines for reporting observational studies. Int. J. Surg..

[33] World Medical Association (WMA) (2017). WMA Declaration of Helsinki—Ethical Principles for Medical Research Involving Human Subjects.

[34] Eliasson A.C., Krumlinde-Sundholm L., Rosblad B., Beckung E., Arner M., Ohrvall A.M., Rosenbaum P. (2006). The Manual Ability Classification System (MACS) for children with cerebral palsy: Scale development and evidence of validity and reliability. Dev. Med. Child Neurol..

[35] Palisano R., Rosenbaum P., Walter S., Russell D., Wood E., Galuppi B. (1997). Development and reliability of a system to classify gross motor function in children with cerebral palsy. Dev. Med. Child Neurol..

[36] Dean E., Dunn W. (2018). Reliability and validity of the child sensory profile 2 Spanish translation. Am. J. Occup. Ther..

[37] Haley S.C., Costel W.J., Dumas H.M., Fragala-Pinkham M.A., Moed R., Kramer J., Ni P., Feng T., Kao Y.-C., Ludlow L.H. (2012). PEDI-CAT Version 1.3.6.: Development, Standardization and Administration Manual.

[38] Thompson S.V., Cech D.J., Cahill S.M., Krzak J.J. (2018). Linking the Pediatric Evaluation of Disability Inventory-Computer Adaptive Test (PEDI-CAT) to the International Classification of Function. Pediatric Phys. Ther..

[39] Dumas H.M., Fragala-Pinkham M.A. (2012). Concurrent validity and reliability of the pediatric evaluation of disability inventory-computer adaptive test mobility domain. Pediatric Phys. Ther..

[40] Dumas H.M., Fragala-Pinkham M.A., Rosen E.L., Lombard K.A., Farrell C. (2015). Pediatric Evaluation of Disability Inventory Computer Adaptive Test (PEDI-CAT) and Alberta Infant Motor Scale (AIMS): Validity and responsiveness. Phys. Ther..

[41] Shore B.J., Allar B.G., Miller P.E., Matheney T.H., Snyder B.D., Fragala-Pinkham M.A. (2017). Evaluating the discriminant validity of the pediatric evaluation of disability inventory: Computer adaptive test in children with cerebral palsy. Phys. Ther..

[42] Shore B.J., Allar B.G., Miller P.E., Matheney T.H., Snyder B.D., Fragala-Pinkham M. (2019). Measuring the reliability and construct validity of the Pediatric Evaluation of Disability Inventory–Computer Adaptive Test (PEDI-CAT) in children with cerebral palsy. Arch. Rhys. Med. Rehabil..

[43] Ravens-Sieberer U., Gosch A., Erhart M., Rueden U., Nickel J., Kurth B.-M., Duer W., Fuerth K., Czemy L., Auquier P. (2006). The KIDSCREEN Questionnaires—Quality of Life Questionnaires for Children and Adolescents—Handbook.

[44] Ravens-Sieberer U., Herdman M., Devine J., Otto C., Bullinger M., Rose M., Klasen F. (2014). The European KIDSCREEN approach to measure quality of life and well-being in children: Development, current application, and future advances. Qual. Life Res..

[45] Kornfeld S., Studer M., Winkelbeiner S., Regenyi M., Boltshauser E., Steinlin M., Swiss Neuropediatric Stroke Group (2017). Quality of life after paediatric ischaemic stroke. Dev. Med. Child Neurol..

[46] Erhart M., Ravens-Sieberer U., Dickinson H.O., Colver A., European S., Groups K. (2009). Rasch measurement properties of the KIDSCREEN quality of life instrument in children with cerebral palsy and differential item functioning between children with and without cerebral palsy. Value Health.

[47] Davis E., Shelly A., Waters E., Davern M. (2010). Measuring the quality of life of children with cerebral palsy: Comparing the conceptual differences and psychometric properties of three instruments. Dev. Med. Child Neurol..

[48] Fritz C.O., Morris P.E., Richler J.J. (2012). Effect size estimates: Current use, calculations, and interpretation. J. Exp. Psychol..

[49] Hopkins W.G., Marshall S.W., Batterham A.M., Hanin J. (2009). Progressive statistics for studies in sports medicine and exercise science. Med. Sci. Sports Exerc..

[50] Soomro N., Kamran B., Bibi R., Ahmed S.I. (2011). Recognizing the sensory abilities in cerebral palsy children. J. Dow Univ. Health Sci. Karachi.

[51] Pavão S.L., Silva F.P., Savelsbergh G.J., Rocha F. (2015). Use of sensory information during postural control in children with cerebral palsy: Systematic review. J. Mot. Behav..

[52] Prakash A., Jerome A., Vaishampayan A. (2007). A preliminary study of the sensory processing abilities of children with cerebral palsy and typical children on the sensory profile. Indian J. Occup. Ther..

[53] Park M.-O. (2017). The relationship between sensory processing abilities and gross and fine motor capabilities of children with cerebral palsy. Korean Soc. Phys. Med..

[54] Auld M.L., Boyd R., Moseley G.L., Ware R., Johnston L.M. (2012). Tactile function in children with unilateral cerebral palsy compared to typically developing children. Disabil. Rehabil..

[55] Østensjø S., Carlberg E.B., Vøllestad N.K. (2003). Everyday functioning in young children with cerebral palsy: Functional skills, caregiver assistance, and modifications of the environment. Dev. Med. Child Neurol..

[56] Mc Manus V., Corcoran P., Perry I.J. (2008). Participation in everyday activities and quality of life in pre-teenage children living with cerebral palsy in South West Ireland. BMC Pediatr..

[57] Michelsen S.I., Flachs E.M., Uldall P., Eriksen E.L., McManus V., Parkes J., Parkinson K.N., Thyen U., Arnaud C., Beckung E. (2009). Frequency of participation of 8–12-year-old children with cerebral palsy: A multi-centre cross-sectional European study. Eur. J. Paediatr. Neurol..

[58] Shelly A., Davis E., Waters E., Mackinnon A., Reddihough D., Boyd R., Reid S., Graham H.K. (2008). The relationship between quality of life and functioning for children with cerebral palsy. Dev. Med. Child Neurol..

[59] Albrecht G.L., Devlieger P.J. (1999). The disability paradox: High quality of life against all odds. Soc. Sci. Med..

[60] Young B., Rice H., Dixon-Woods M., Colver A.F., Parkinson K.N. (2007). A qualitative study of the health-related quality of life of disabled children. Dev. Med. Child Neurol..

[61] Alsem M.W., Ketelaar M., Verhoef M. (2013). The course of health-related quality of life of preschool children with cerebral palsy. Disabil. Rehabil..

[62] Baranek G.T., Chin Y.H., Hess L.M., Yankee J.G., Hatton D.D., Hooper S.R. (2002). Sensory processing correlates of occupational performance in children with fragile X syndrome: Preliminary findings. Am. J. Occup. Ther..

[63] White B.P., Mulligan S., Merrill K., Wright J. (2007). An examination of the relationships between motor and process skills and scores on the sensory profile. Am. J. Occup. Ther..

[64] Hagert E. (2010). Proprioception of the wrist joint: A review of current concepts and possible implications on the rehabilitation of the wrist. J. Hand Ther..

[65] Auld M.L., Boyd R.N., Moseley G.L., Ware R.S., Johnston L.M. (2012). Impact of tactile dysfunction on upper-limb motor performance in children with unilateral cerebral palsy. Arch. Phys. Med. Rehabil..

[66] Gordon A.M., Duff S.V. (1999). Fingertip forces during object manipulation in children with hemiplegic cerebral palsy. I: Anticipatory scaling. Dev. Med. Child Neurol..

[67] Gordon A.M., Duff S.V. (1999). Relation between clinical measures and fine manipulative control in children with hemiplegic cerebral palsy. Dev. Med. Child Neurol..

[68] Kinnucan E., Van Heest A., Tomhave W. (2010). Correlation of motor function and stereognosis impairment in upper limb cerebral palsy. J. Hand Surg. Am..

[69] Klingels K., Demeyere I., Jaspers E., De Cock P., Molenaers G., Boyd R., Feys H. (2012). Upper limb impairments and their impact on activity measures in children with unilateral cerebral palsy. Eur. J. Paediatr. Neurol..

[70] Krumlinde-Sundholm L., Eliasson A.C. (2002). Comparing tests of tactile sensibility: Aspects relevant to testing children with spastic hemiplegia. Dev. Med. Child Neurol..

[71] Sakzewski L., Ziviani J., Boyd R., Neurology C. (2010). The relationship between unimanual capacity and bimanual performance in children with congenital hemiplegia. Dev. Med. Child Neurol..

[72] Dunn W. (1997). The impact of sensory processing abilities on the daily lives of young children and their families: A conceptual model. Infants Young Child..

[73] Adams J.N., Feldman H.M., Huffman L.C., Loe I.M. (2015). Sensory processing in preterm preschoolers and its association with executive function. Early Hum. Dev..

[74] Hahn N., Foxe J.J., Molholm S. (2014). Impairments of multisensory integration and cross-sensory learning as pathways to dyslexia. Neurosci. Biobehav. Rev..

[75] Mailloux Z., Mulligan S., Roley S.S., Blanche E., Cermak S., Coleman G.G., Bodison S., Lane C.J. (2011). Verification and clarification of patterns of sensory integrative dysfunction. Am. J. Occup. Ther..

