# Supplementary material for: Sensory Processing, Functional Performance and Quality of Life in Unilateral Cerebral Palsy Children: A Cross-Sectional Study
Source: Int J Environ Res Public Health. 2020 Sep 28;17(19):7116. doi: 10.3390/ijerph17197116 (PMC7579654; doi:10.3390/ijerph17197116)
Supplement: Supplementary file 1 [file ijerph-17-07116-s001.pdf]

Table S1. Correlations between CSP-2 and PEDI-CAT domains.

	All (TD + UCP) n (49)				TD n (23)				UCP n (26)			
CSP-2	DA	M	S/C	R	DA	M	S/C	R	DA	M	S/C	R
Seeking	-0.05	-0.06	-0.29*	-0.08	-0.23	-0.18	-0.32	0.02	0.05	0.03	-0.23	-0.13
Avoidance	-0.44**	-0.41**	-0.25	-0.23	-0.48*	-0.33	-0.37	-0.43*	-0.07	-0.16	-0.01	-0.06
Sensitivity	-0.34*	-0.32*	-0.32*	-0.32*	-0.43*	-0.19	-0.42*	-0.33	-0.09	-0.18	-0.17	-0.3
Registration	-0.58***	-0.48***	-0.34*	-0.13	-0.42*	-0.15	-0.43*	-0.04	-0.14	-0.21	-0.06	-0.04
Auditory	-0.24	-0.17	-0.23	-0.37**	-0.2	0.03	-0.16	-0.46*	0.04	-0.04	-0.14	-0.31
Visual	-0.04	-0.1	-0.31*	-0.12	-0.26	-0.2	-0.37	-0.09	0.02	-0.02	-0.27	-0.14
Touch	-0.22	-0.16	-0.18	0.04	-0.35	-0.07	-0.28	0.22	-0.07	-0.06	-0.02	-0.07
Movement	-0.19	-0.12	-0.18	-0.08	-0.1	0.13	-0.14	0.13	0.01	-0.05	-0.09	-0.21
BodyPos	-0.70***	-0.67***	-0.41**	-0.13	-0.46*	-0.37	-0.58**	0.04	-0.39*	-0.39*	-0.21	-0.05
Oral	-0.36*	-0.41**	-0.27	-0.31*	-0.31	-0.44*	-0.38	-0.32	-0.06	-0.12	-0.05	-0.26
Conductual	-0.08	-0.03	-0.01	-0.03	-0.19	0.12	-0.16	0.01	0.11	-0.01	0.16	-0.03
Social-Emo	-0.46***	-0.44**	-0.27	-0.11	-0.43*	-0.31	-0.29	-0.28	-0.12	-0.23	-0.02	0.08
Attentional	-0.27	-0.24	-0.29*	-0.12	-0.42*	-0.19	-0.43*	-0.18	0.14	0.06	0.01	0.04

CSP-2: Child Sensory Profile 2; PEDI-CAT: Pediatric Evaluation of Disability Inventory – Computer Adaptive Test; TD: Typical Development; UCP: Unilateral Cerebral Palsy; SD: Standard Deviation; BodyPos: Body Position; Social-Emo: Social-Emotional; DA: Daily Activities; M: Mobility; S/C: Social/Cognitive; R: Responsibility. Pearson correlation´s coefficients; *** = p<0.001; ** = p<0.01; * p< 0.5.

Table S2. Correlations between CSP-2 and KD-27 and KD-10 domains.

	All (TD+ UCP) n (36)						TD n (16)						UCP n (20)					
	KS-27					KS10	KS-27					KS10	KS-27					KS10
CSP-2	PW	PCW	P&A	SS&P	SE	GI	PW	PCW	P&A	SS&P	SE	GI	PW	PCW	P&A	SS&P	SE	GI
Seeking	0.07	-0.17	0.01	-0.24	-0.24	-0.15	0.33	0.10	0.07	-0.50*	-0.23	-0.01	-0.18	-0.24	0.10	-0.07	-0.18	-0.31
Avoidance	-0.06	-0.09	0.45**	-0.11	-0.20	-0.23	0.30	0.33	0.57*	0.04	-0.05	-0.19	-0.11	-0.36	0.30	-0.27	-0.39	-0.11
Sensitivity	-0.07	-0.17	0.20	-0.13	-0.29	-0.18	0.29	0.18	0.56*	-0.17	-0.26	-0.06	-0.19	-0.34	-0.01	-0.14	-0.43	-0.14
Registration	-0.25	-0.14	0.19	-0.17	0.02	-0.15	0.29	0.16	0.11	-0.53*	-0.04	0.08	-0.20	-0.44	0.05	-0.15	-0.20	0.08
Auditory	-0.05	-0.13	0.11	-0.15	-0.52***	-0.12	0.40	0.31	0.38	0.08	-0.50	-0.25	-0.28	-0.35	-0.11	-0.25	-0.63**	-0.02
Visual	-0.06	-0.17	-0.11	-0.39*	-0.42**	-0.06	-0.02	-0.25	-0.22	-0.53*	-0.16	0.14	-0.33	-0.10	0.08	-0.36	-0.51*	-0.26
Touch	0.16	-0.17	0.08	-0.13	-0.10	0.06	0.46	0.02	0.11	-0.32	0.17	0.17	0.11	-0.34	-0.07	-0.01	-0.46*	0.00
Movement	0.06	-0.03	0.02	-0.06	0.01	-0.09	0.47	0.21	-0.01	-0.24	-0.20	-0.16	-0.18	-0.15	0.04	0.01	0.10	-0.03
BodyPos	-0.40*	-0.09	0.29	-0.08	0.09	-0.25	-0.07	0.03	0.18	-0.31	-0.18	-0.03	-0.11	-0.27	0.21	0.01	0.00	-0.18
Oral	-0.18	-0.12	0.36*	-0.03	0.00	-0.32	-0.13	-0.04	0.39	-0.17	-0.33	-0.22	-0.14	-0.21	0.35	-0.02	-0.06	-0.32
Conductual	0.06	-0.27	0.04	-0.19	-0.11	0.17	0.40	0.21	0.11	-0.42	-0.14	0.01	-0.18	-0.64**	-0.06	-0.03	-0.15	0.34
Social-Emo	-0.13	-0.12	0.49**	-0.04	-0.01	-0.24	0.24	0.31	0.56*	0.29	0.20	-0.17	-0.20	-0.49*	0.37	-0.25	-0.20	-0.15
Attentional	-0.06	-0.12	0.17	-0.29	-0.22	-0.17	0.26	0.23	0.28	-0.38	-0.22	-0.14	-0.19	-0.33	0.08	-0.26	-0.27	-0.13

CSP-2: Child Sensory Profile 2; TD: Typical Development; UCP: Unilateral Cerebral Palsy; SD: Standard Deviation; BodyPos: Body Position; Social-Emo: Social-Emotional; PW: Physical Well-Being; PCW: Psychological Well-Being; P&A: Parents & Autonomy; SS&P: Social Support & Peers; SE: School Environment; GI: General Index; Pearson correlation´s coefficients: *** = p<0.001; ** = p<0.0; * p< 0.05.
